# Deconstructing and Assessing Knowledge and Awareness in Public Health Research

**DOI:** 10.3389/fpubh.2017.00194

**Published:** 2017-08-07

**Authors:** Robert Trevethan

**Affiliations:** ^1^Independent academic researcher and author, Albury, NSW, Australia

**Keywords:** knowledge, awareness, public health, health beliefs, health behavior, healthcare, epidemiology

## Abstract

When people’s knowledge and awareness are the subject of public health research, the meanings applied to the words knowledge and awareness are often unclear. Although frequently used interchangeably without that being problematic, these words sometimes appear to have different intended meanings but those meanings are not made explicit or, despite the meanings having been made explicit, they are not adhered to. It is necessary to overcome obscurities when knowledge and awareness are intended to represent different domains. This occurs when they are compared with each other; it also occurs when knowledge and awareness are assessed separately in relation to such variables as health behavior; physical, psychological, or socioeconomic statuses; gender; age; and ethnic backgrounds. For those particular research ventures, recommendations are made that knowledge be used to refer to information that is, to a greater or lesser extent, detailed and factual, and that awareness be associated with information that is personally relevant. Some suggestions are made, and issues are raised, about how the psychometric foundations for each of those two domains might be established prior to use in empirical research. Adopting the recommendations and suggestions made in this article provides opportunities for greater conceptual and empirical clarity and success.

## Introduction

The words knowledge and awareness are sometimes juxtaposed in public health research literature. When they appear, these two words often refer to the general information and perceptions that people possess and exhibit, but specific reference is sometimes made to their own health and to healthcare providers’ knowledge and awareness. There is a broad range of research and theoretical literature within which the words knowledge and awareness have been used. Topics include, but are not restricted to, communicable diseases such as dengue fever (1), malaria (2), and sexually transmitted diseases (3–7); non-communicable diseases such as Alzheimer’s disease (8), Parkinson’s disease (9), kidney disease (10), and cardiovascular diseases (11–19); diabetes (20, 21); cancer, including oral oncology (22, 23); glaucoma (24, 25); general dental health (26); palliative care (27); food and drug interactions (28, 29); healthy lifestyles (30); practices of healthcare providers (31–34); the existence and availability of healthcare services (5); and healthcare insurance (35).

The meanings that are assigned to the words knowledge and awareness are not consistent within this broad literature. Often the lack of definitional sharpness is unproblematic and the text of a publication flows smoothly without any definitional clarity appearing to be necessary (9, 12, 14, 16–19, 23–25, 28, 29, 34, 35). This is particularly the case when awareness is obviously, and consistently, used to refer to people having generalized or diffuse knowledge about the existence of something—as if they had been asked a question such as “Have you ever heard of Parkinson’s disease?” to which an answer can be simply Yes or No with the expectation that even an affirmative answer need not be associated with any further knowledge. This has been referred to by McCallum et al. (5) as general awareness, and occasionally an explicit definition concerning it is provided by researchers (5, 24, 25). Sometimes, however, this particular kind of awareness is clearly implied but any distinction between it and knowledge is not sustained (2, 6, 7, 33). For example, Dhawan et al. (2) referred to 95.7% of their respondents having heard of malaria and therefore being aware of it, but they presented these findings under a heading referring to malaria-related knowledge. Any difference between knowledge and awareness is blurred under these circumstances.

Furthermore, for some researchers (1, 4, 10, 15, 20, 30, 31), the word awareness seems to be used almost haphazardly. In some places, it seems to represent something conceptually distinct from knowledge, in other places it seems to be completely synonymous with knowledge, and in still other places it is seemingly added for good measure with no specific meaning either intended or intended to remain constant. Jeelani et al. (1), for example, used the words knowledge and awareness in the title of their article, omitted awareness as a target within the aims of their research (where they substituted attitudes for awareness), reintroduced awareness as a specific target when describing their study design and in some of their analyses but seem to regard awareness as a subcomponent of knowledge in other analyses, and focused on knowledge, not awareness, in their most substantive analyses.

The above inconsistencies are not atypical. Although they might be dismissed as minimally frustrating, and perhaps in some cases as trivial editorial oversights on the part of authors, distinct problems arise if knowledge and awareness are intended to demarcate clearly identifiable and distinct conceptual and empirical domains. One area in which this might occur is in research about whether or not knowledge and awareness—whatever those words might mean—parallel each other, for example, whether knowledge about obesity is related to awareness about obesity, or whether greater awareness about obesity leads to greater knowledge about it. Problems also arise if there is an intention to relate knowledge and awareness, independently, to other variables including health-relevant behavior; physical, psychological, or socioeconomic statuses; gender; age; or ethnic backgrounds. Relevant research questions might focus on whether awareness is a sufficient determinant, or whether their knowledge is a stronger determinant, when encouraging people to adopt behavior that avoids obesity.

Research questions such as these, and there are many more, reveal that at times there is an obvious need for definitional clarity concerning knowledge and awareness. In this brief article, suggestions are made about achieving that clarity, beginning with an exploration of definitions that are provided in dictionaries. These definitions are helpful because they reveal the foundations of conceptual problems that typify some research, but they also provide an avenue for achieving a solution when a distinction between knowledge and awareness is needed. This article concludes with some fundamental suggestions about measurement of knowledge and awareness as separate domains.

## Dictionary Clarifications, Obfuscations, and Solutions

For the word knowledge, primary entries in five online dictionaries (36–40) provide definitions that meet commonly held understandings and appear to be implicitly accepted by many researchers. Those primary entries are shown in the central column of Table 1. Together, they indicate that knowledge comprises information that is acquired from authoritative external sources and that can therefore, presumably, be regarded as factual in nature. In the context of health, this information often falls within the purview of public health and epidemiology. It includes, where appropriate, knowledge about prevalence, etiology, risk factors, prevention, transmission, pathophysiology, symptomatology, progression, recommended action in the presence or event of specific health problems, treatment, precautions, sequelae, existence and availability of healthcare services, and patient rights. It might, therefore, be anticipated that researchers who investigate knowledge, which has been referred to by McCallum et al. (5) as specific knowledge, will focus on variables of this kind. Some researchers (8, 11, 12) clearly do so. For them, it is evident that knowledge refers solely to specific information that is factual in nature.

**Table 1:** Dictionary entries concerning knowledge.

Dictionary	Initial/primary entry	Selected subsequent entries and synonyms
Cambridge Dictionary	Understanding of or information about a subject that you get from experience or study	Awareness of something: the state of being aware of something
Dictionary.com	Acquaintance with facts, truths, or principles	Awareness, as of a fact or circumstance
The Free Dictionary	The state or fact of knowing	Awareness, consciousness, or familiarity gained by experience or learning
Merriam-Webster Dictionary	Information, understanding, or skill that you get from experience or education	Awareness of something: the state of being aware of something
Oxford Dictionaries	Facts, information, and skills acquired through experience or education	Awareness or familiarity gained by experience of a fact or situation

There are obvious problems, however, with the word awareness, particularly concerning how it might be differentiated from knowledge. Some of the difficulty in distinguishing between knowledge and awareness is understandable following an inspection of the subsidiary dictionary definitions for knowledge, a number of which are provided in the final column of Table 1. There it can be seen that, for all five dictionaries, the word awareness predominates as a secondary way of defining knowledge, thus demonstrating a manifest overlap of meanings between the two words.

An attempt to distinguish between knowledge and awareness remains difficult when the primary definitions for awareness are sought from all five dictionaries. These primary definitions are provided in the central column of Table 2, where it can be seen that the words knowing and knowledge not only feature in defining awareness but are usually predominant.

**Table 2:** Dictionary entries concerning awareness.

Dictionary	Initial/primary entry	Selected subsequent entries and synonyms
Cambridge Dictionary	Knowing that something exists, or understanding of a situation or subject at the present time based on information or experience	Acquaintance, consciousness, knowledge
Dictionary.com	The state or condition of being aware; having knowledge; consciousness	Attentiveness, apprehension, consciousness, familiarity, mindfulness
The Free Dictionary	Having knowledge or discernment of something	Acquaintance with, attention to, awake, consciousness of, knowingness, mindfulness of, self-awareness, sense of danger, sensibility to. Archaic: vigilant, watchful
Merriam-Webster Dictionary	Knowing that something (such as a situation, condition, or problem) exists. Archaic: watchful, wary	Awake, conscious, vigilance
Oxford Dictionaries	Knowledge or perception of a situation or fact	Appreciation, consciousness, familiarity

A clear distinction between knowledge and awareness therefore appears to be elusive, and awareness certainly appears to be strongly associated with knowledge. As a result, it is not surprising that many researchers, whether intentionally or not, use the two words as if they are semantically interchangeable, and other researchers use the words in ways that are intended to be different but the difference is not made explicit or is not sustained.

**Figure 1:**
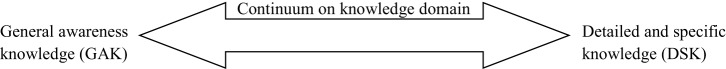
Representation of knowledge as a continuum on a single domain.

Partial resolution can be achieved by regarding the McCallum et al. (5) notion of specific knowledge as sitting at the high end of a continuum based on information specificity and accuracy, with the same researchers’ notion of general awareness sitting at the low end of the same continuum to represent people having no, or very little, knowledge about a topic at hand. According to this perspective, knowledge and awareness are not qualitatively different. They simply occupy opposite positions on a single continuum—a knowledge continuum. This appears to be the usual way in which the words knowledge and awareness are used in relation to each other, although that is almost never made explicit. It is depicted, with suggested abbreviations, in Figure 1, where the McCallum et al. *general awareness* sits at the lower end of the continuum, and the McCallum et al. *detailed and specific knowledge* (DSK) sits at the higher end. This might be referred to as a *knowledge domain*.

**Figure 2:**
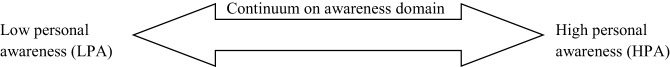
Representation of awareness as a continuum on a single domain.

The possibility of drawing a distinction between knowledge and awareness from a different perspective—as comprising separate, distinct, domains rather than being opposite points of a single continuum—emerges from inspection of entries in the final column of Table 2. There, a number of secondary definitions for awareness include words such as acquaintance, attentiveness, attention to, mindfulness, and self-awareness—and even apprehension, sense of danger, and vigilance, suggesting that there might be etymological links with awareness in words such as beware (to be on one’s guard) and wary (being watchful or cautious). All of these words are differently nuanced among themselves, but without exception they contain strong elements of personalization, a self-focus, and personal familiarity, that are not present in the more impersonal, detached, and (allegedly) factual nature of information that is typically associated with the word knowledge. This reveals the opportunity for drawing a distinction between the words knowledge and awareness that differs from the specific knowledge/general awareness distinction referred to above—valuable as that distinction might be at times. The proposal being put forward here is that there is a particular kind of awareness—one that is personal in nature—that could be regarded in some contexts as the specific, and different, referent when the word awareness is used. More specifically, this kind of awareness might be regarded as referring to a domain that spans the extent of personal engagement or concern. It is depicted, also with suggested abbreviations, in Figure 2, where low personal awareness (LPA) sits at one end of a continuum, and high personal awareness (HPA) sits at the other. This might be referred to as an *awareness domain*.

If theorists and researchers accept the existence of the above two domains, in particular the two different types of awareness, they could avoid much conceptual and empirical ambiguity when knowledge and awareness need to be regarded as distinctly different from each other.

## Consolidating the Nature of Personalized Awareness

Having identified a specific kind of awareness, one that has a strong personal element, it becomes readily possible to identify domains within health research to which it would refer. These include attentive self-perceptions about conditions related to health, whether accurate or not (e.g., “I think I am putting on too much weight”), awareness about definitely having, or definitely not having, a particular health status (e.g., “I am aware that I have/do not have diabetes”), apprehension about prospective health problems that might arise from genetic susceptibilities (e.g., “My mother had/has breast cancer”), strength of personal concerns about one’s own health status (e.g., “I worry a lot/avoid thinking about whether I might be HIV positive”), heightened sensitivity because of the health status of a close relative or acquaintance (e.g., “My husband/good friend/neighbor has had bowel cancer”), and awareness about one’s own need to engage in health-enhancing behavior or to avoid health-damaging behavior (e.g., “I should avoid doing things that put me at risk of poor dental health”).

Given its personal focus, this kind of awareness is conceptually distinct from the kind of general awareness about the mere existence of something—the awareness that lies at the lower end of the knowledge continuum proposed earlier in this article and as shown in Figure 1—and some researchers obviously employ this personalized perspective in a deliberate way when referring to awareness. Dokubo et al. (3), for example, assessed whether people were aware of their own HIV status; Crouch (13), while investigating women’s awareness and knowledge concerning heart disease, referred to awareness in the form of general awareness (at what is proposed above as the low end of the knowledge continuum), but also related awareness to the women’s sensitivity concerning their *own* risk of heart disease—i.e., the personalization of risk.

## Basic Psychometric Suggestions and Considerations

If there is to be a general awareness knowledge (GAK)/DSK domain that represents *knowledge*, as shown in Figure 1, there needs to be an effective way of combining general awareness and knowledge within a single dimension in research situations. This need not be difficult to achieve. For example, Medieros and Ramada (6) asked university students whether they had ever heard of human papillomavirus (HPV). If the students had not heard of this virus (i.e., were not even aware of it), they were moved to the next major section of the questionnaire. If, however, the students *had* heard about HPV they were asked subsequent, more specific, questions to assess the extent of their knowledge about it. In that way, both GAK and DSK could be assessed by questions that spanned a single knowledge continuum, with students who had never heard of the virus obtaining the lowest score on that continuum (at the GAK pole; refer to Figure 1) and other students obtaining increasingly higher scores (toward the DSK pole) the more they knew.

The same process and outcomes could occur for the personal awareness domain, shown in Figure 2, ranging from LPA to HPA. If respondents indicated that they were not aware about a particular topic in any ways that were personally relevant to them, they would be given the lowest score on that domain (at the LPA pole to represent no personal awareness or concern), and there would be no need to ask further questions to tap that domain. The more that, in light of further questioning, respondents were aware about their own personal association with the particular topic of interest, the higher their score would be for this kind of awareness (thus moving toward the HPA pole that represents high personal awareness).

Anticipating and applying these straightforward psychometric strategies could generate much-needed clarity in some conceptual and empirical contexts.

Procedures for developing, as well as for establishing, the psychometric credentials of instruments in health research (41–43) are beyond the intended scope of this article. However, in order to avoid giving an undue sense of resolution based simply on the above recommendations, and to increase the opportunities for sharpening the distinction between knowledge and awareness that is being proposed here, at least when doing so is considered necessary, some issues concerning the kind of responses that are elicited from respondents, and the way those responses are scored, will be dealt with briefly because they often seem to have received insufficient attention within research concerning people’s knowledge and awareness. A number of issues require careful thought on the part of instrument developers, and the most effective strategies are not always obvious or easily implemented.

With regard to the validity of responses that are elicited from respondents, issues arise about whether to offer open-ended (recall-dependent) or fixed (recognition-dependent) options, and, if the latter, how many and what kind of options should be provided. For many researchers (2, 4, 6, 11, 15, 20, 26, 28–30), assessing knowledge has been attempted by compiling a list of items (e.g., *Diabetes is incurable*) to which respondents are offered two or three choices (e.g., *Correct/Incorrect*, or *True/False/I don’t know*), and the correct responses are added to obtain a single score. Although this recall-dependent strategy is usually regarded as acceptable, Hunt [(44), p. 100] has suggested that it has “serious deficiencies” and that assessing a test-taker’s certainty about each item would produce more valid total scores. Other strategies are worth pursuing. They include use of tailored Likert-type options that are deemed most suitable for particular items.

Possibilities and issues related to *scoring* also need to be addressed carefully to maximize validity. For example, in some situations a response of *Yes* might be accorded a score of 1 whereas responses of *No* and *Don’t know* might both attract scores of zero. However, in other situations, that scoring strategy might not capture a construct adequately or appropriately. In questions assessing knowledge, for example, validity might be enhanced if higher scores were assigned to correct responses on questions that refer to more important aspects of a construct and lower scores to less important aspects, as done by Sarumathi et al. (26). Furthermore, within the context of personal awareness, responses of *Yes* and *No*, if based on professional diagnoses, could well be scored similarly (they both indicate concrete and informed personal awareness) and they could be scored higher than responses of *Don’t know*. It might be advisable, however, to allocate a higher score to a *Yes* response than to a *No* response under some circumstances involving personal awareness, but careful thought would need to be exercised concerning that strategy in concert with the aims of a particular piece of research.

## Conclusion

It is obvious that defining, writing about, and measuring knowledge and awareness, if those two words are to be meaningful and useful in public health research, requires more care than initially seems necessary. Applying that care should not only avoid conceptual confusion and misguided quantification where they might otherwise exist, but also create improved prospects for more definitive and refined research findings.

## Author Contributions

RT conceived of, conducted the research for, and wrote the complete manuscript.

## Conflict of Interest Statement

The author declares that this research was conducted in the absence of any commercial or financial relationships that could be construed as a potential conflict of interest.
